# An adaptive current–voltage step size solver for accurate and efficient modeling of solar photovoltaic modules

**DOI:** 10.1371/journal.pone.0359796

**Published:** 2026-10-06

**Authors:** Nsulwa John Mlazi, Baraka Kichonge

**Affiliations:** 1 Department of Applied Sciences and Social Studies, Arusha Technical College, Arusha, Tanzania; 2 School of Materials, Energy, Water and Environmental Sciences (MEWES), The Nelson Mandela African Institution of Science and Technology (NM-AIST), Arusha, Tanzania; 3 Department of Mechanical Engineering, Arusha Technical College, Arusha, Tanzania; Dr Shakuntala Misra National Rehabilitation University, INDIA

## Abstract

Accurate modeling of photovoltaic (PV) modules is critical for system design, energy yield prediction, and maximum power point tracking (MPPT). Conventional single-diode models (SDM) provide a balance between simplicity and physical realism but require iterative numerical methods to solve the implicit current–voltage (I–V) equation, often leading to slow convergence and reduced stability under dynamic conditions. This paper introduces a novel adaptive current–voltage step-size solver (AIVSS) that dynamically adjusts both voltage and current increments based on the local slope of the I–V curve, thereby improving numerical accuracy and convergence efficiency. The proposed method was validated through 500 simulations under standard test conditions (STC), partial shading, high temperature, and dynamic irradiance, and was compared with the conventional adaptive voltage-step solver (AVSS). Performance was evaluated using mean absolute error (MAE), root mean square error (RMSE), iteration count, and computation time. Results show that AIVSS reduced the average iteration count by 35–40%, decreased the average MAE from 6.15 W to 2.18 W, and reduced the average RMSE from 7.68 W to 2.85 W across all operating conditions. In addition, the proposed method achieved faster convergence and smoother dynamic response under rapidly changing irradiance. These findings confirm the strong potential of AIVSS for real-time PV system simulation and MPPT applications, providing a robust and computationally efficient tool for accurate PV module characterization.

## Introduction

The growing global demand for renewable energy has accelerated the deployment of solar PV systems as a clean, sustainable, and reliable electricity source. Accurate modeling of PV modules is essential for system design optimization, energy yield prediction, and the development of efficient MPPT algorithms [1–3]. The nonlinear I–V characteristics of PV modules are strongly influenced by irradiance, temperature, and intrinsic device parameters, making precise representation vital for both simulation and real-time control. Accurate I–V modeling directly impacts MPPT efficiency and the performance of embedded PV controllers, highlighting the importance of robust numerical solvers in PV applications [4–6].

The single-diode model (SDM) has been widely adopted due to its balance of simplicity and physical accuracy [2,7]. In this model, the I–V relationship is implicit and nonlinear, necessitating iterative numerical techniques for solution. Traditional approaches, such as the Newton–Raphson (NR) method and Lambert W function-based formulations [5,8], provide high accuracy but often encounter convergence challenges or incur high computational cost across wide voltage ranges. To address these issues, several enhanced numerical schemes have been developed. Modified or damped NR methods improve convergence stability near the maximum power point [9,10], while metaheuristic optimization techniques including particle swarm optimization (PSO), genetic algorithms (GA), and artificial bee colony (ABC) methods enable precise parameter extraction and curve fitting [11,12]. Although effective, these approaches are typically computationally intensive, limiting their practical applicability for real-time or embedded PV systems.

Recent research has focused on reducing iteration counts and simulation time. For instance, Azure et al. [13] proposed a Broyden-like iterative approach with faster convergence than standard Newton-Raphson method, while approximate SDM formulations can reduce simulation time by up to 30% without sacrificing I–V curve accuracy. Hybrid analytical–numerical approaches further improve agreement with manufacturer datasheet values and minimize MPP errors [6]. Nonetheless, comparative studies indicate that nonlinear regions near the MPP remain challenging for both analytical and numerical models [6], emphasizing the need for more efficient and accurate solvers.

Parallel developments in MPPT research demonstrate that variable or adaptive step-size techniques improve computational efficiency and reduce oscillations under dynamic conditions [13]. Similarly, adaptive numerical solvers in differential equation and control applications dynamically vary step sizes according to local slope or error [14]. While adaptive voltage-step methods have been applied to PV modeling, they focus solely on voltage increments, leaving current adaptation unaddressed. Consequently, many existing approaches still rely on uniformly spaced voltage sampling, which is inefficient near highly nonlinear regions such as the MPP or open-circuit voltage ($V_{oc}$). This limitation motivates the exploration of dual-step adaptation strategies that simultaneously adjust both voltage and current increments for improved performance.

Despite the advancements in adaptive solvers across computational domains, simultaneous voltage-current step adaptive in static PV modeling has received limited attention. Existing adaptive voltage-step approaches [15–18] improve convergence but do not fully ensure numerical stability or accuracy under nonlinear and dynamic operating conditions.

To address this gap, this study introduces a novel adaptive current–voltage step-size solver (AIVSS) that adjusts both voltage and current increments according to the local slope of the I–V curve. Unlike existing adaptive solvers that adjust only voltage increments or rely solely on reactive adaptation, the proposed AIVSS uniquely integrates bidirectional step adaptation with predictive slope-based correction, enabling simultaneous control of both electrical variables. The proposed solver enhances computational efficiency by minimizing redundant calculations in quasi-linear regions while improving numerical accuracy near the MPP and $V_{oc}$. It provides a lightweight yet effective enhancement to conventional PV simulation techniques, supporting faster and more precise real-time PV and MPPT applications.

The primary contribution of this work lies in improving numerical PV module modeling through an adaptive dual-step solver. While the method can support MPPT applications, it does not replace MPPT algorithms but rather enhances their accuracy through improved I–V curve estimation. The rest contributions of this paper are summarized as follows. This work introduces a novel AIVSS that dynamically and simultaneously regulates both voltage and current increments to improve tracking efficiency. It further enhances system performance by incorporating predictive correction terms, which significantly improve convergence stability and suppress oscillations, especially in the vicinity of the maximum power point (MPP). In addition, the proposed method is extensively validated under both steady-state and dynamic conditions, including challenging scenarios such as partial shading and rapidly varying irradiance. Finally, experimental results obtained from a real photovoltaic module confirm the practical effectiveness of the approach, demonstrating improved accuracy, robustness, and overall performance compared to conventional methods.

Finally, the remainder of the paper is organized as follows: The section of Mathematical Model of Solar PV Module presents the mathematical formulation of the SDM PV model. Adaptive Step Numerical Solver section describes the adaptive current–voltage step-size numerical solver and implementation algorithm. Simulation Setup section outlines the setup of simulation and parameters. Results and Discussion section discusses results and performance evaluation, while Conclusion section concludes the paper with key findings and directions for future research.

## Mathematical model of solar PV module

Accurate modeling of solar PV modules is critical for predicting performance, designing efficient systems, and implementing MPPT algorithms. The mathematical representation of a PV module captures the nonlinear relationship between voltage, current, and environmental factors such as irradiance and temperature. Among the various modeling approaches, the SDM is widely favored due to its balance of physical realism and computational simplicity. This section presents the SDM, describes its equivalent circuit, defines the governing I–V equation, and explains the temperature and irradiance dependence of key parameters, which are essential for realistic simulation and evaluation of numerical solvers.

### Single-diode equivalent circuit

The SDM is the most widely adopted representation of a PV cell or module, providing an optimal balance between modeling accuracy and computational simplicity [7,19]. As illustrated in Fig 1, the model consists of a photocurrent source ($I_{ph}$), a diode representing the p–n junction, a series resistance ($R_{s}$), and a shunt resistance ($R_{sh}$) that accounts for leakage currents.

**Fig 1:**
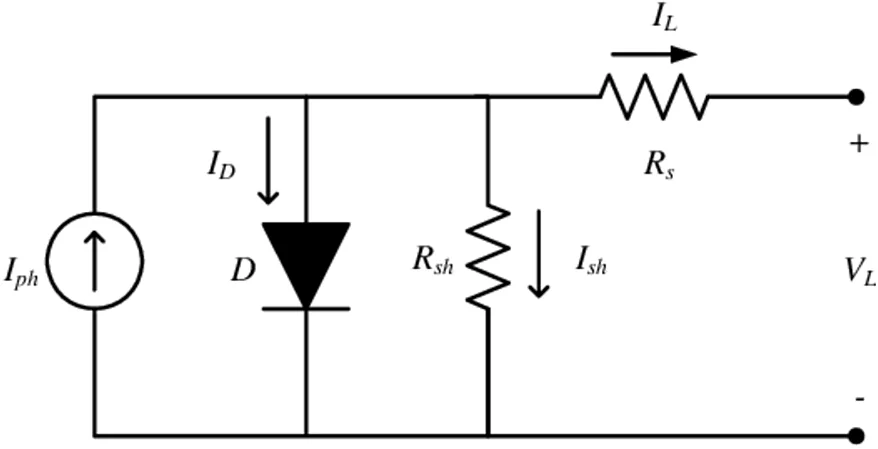
Electrical equivalent circuit of the single-diode PV model.

The I–V relationship of the SDM is expressed as [20–24]:


$$\begin{array}{c} I=I_{ph}-I_{0}\left[\exp\left(\frac{V+IR_{s}}{nV_{t}}\right)-1\right]-\frac{V+IR_{s}}{R_{sh}} \end{array}$$ (1)

where *I*_0_ is the diode reverse saturation current, *n* is the diode ideality factor, and $V_{t}=kT/q$ is the thermal voltage. Due to the implicit nature of Eq. (1), iterative numerical solvers are required to compute the I–V curve [25].

### Parameter dependence

The SDM parameters ($I_{ph}$, *I*_0_, $R_{s}$, $R_{sh}$, and *n*) are functions of operating temperature and irradiance. The photocurrent $I_{ph}$ is approximately proportional to solar irradiance *G*, while the saturation current *I*_0_ increases exponentially with temperature [26,27]. These dependencies can be modeled as:


$$\begin{array}{c} I_{ph}=\left(\frac{G}{G_{STC}}\right)\left[I_{ph,STC}+\alpha_{I}(T-T_{STC})\right] \end{array}$$ (2)

$$\begin{array}{c} I_{0}=I_{0,STC}{\left(\frac{T}{T_{STC}}\right)}^{3}\exp\left[\frac{qE_{g}}{nk}\left(\frac{1}{T_{STC}}-\frac{1}{T}\right)\right] \end{array}$$ (3)

where:

- $E_{g}$ is the semiconductor bandgap energy (1.12 eV for silicon),
- $G_{STC}=1000\;W/m^{2}$ and $T_{STC}=298.15\;K$ denote standard test conditions,
- $\alpha_{I}$ is the temperature coefficient of photocurrent.

The temperature coefficient of voltage (β) was not explicitly incorporated in the present model because the primary objective of this study was to evaluate the numerical performance of the proposed solver rather than to refine temperature-dependent parameter extraction. Since both AVSS and AIVSS were evaluated under the same modeling assumptions, the comparative accuracy of the proposed method remains valid.

Having established the mathematical model of the PV module and characterized its parameters, the next step is to solve the implicit I–V equation efficiently. Traditional solvers such as Newton–Raphson may face convergence challenges, motivating the development of the proposed AIVSS. For this study, simulations are conducted under STC to isolate and evaluate the performance of the numerical solver. Environmental factors such as irradiance and temperature are therefore held constant, with manufacturer-provided parameters (Table 1) used as fixed inputs.

**Table 1:** Descriptions of the model parameters at $1000\;W/m^{2}$ and $25^{\circ }$C.

Parameters	Unit
Maximum Power ($P_{max}$)	300 Wp
Maximum Variation	± 5%
Open-Circuit Voltage ($V_{oc}$)	41.25 V DC
Short-Circuit Current ($I_{sc}$)	9.54 A DC
Module Dimensions	1640 × 992 × 35 mm
Module Weight	18.1 kg
Cell Type	Polycrystalline
Model	Chloride CHL285P
Number of Cells	60
Maximum System Voltage	1000 V DC

## Adaptive step numerical solver

### Overview

Accurate modeling of solar PV modules requires solving the implicit nonlinear I–V relationship of the single-diode model. Traditional Newton–Raphson (NR) solvers provide quadratic convergence but rely on fixed voltage increments (ΔV), which can result in redundant computations in quasi-linear regions and insufficient resolution near highly nonlinear regions such as the MPP or open-circuit voltage ($V_{oc}$) [7,25]. Adaptive step strategies improve computational efficiency while maintaining accuracy by adjusting the step size according to local nonlinearity.

### Newton–Raphson solver

The Newton–Raphson method iteratively solves the implicit I–V equation of the single-diode PV model in Eq. 1

The NR iteration updates the current as [28–32]:


$$\begin{array}{c} I^{\left(j+1\right)}=I^{\left(j\right)}-\frac{f(I^{\left(j\right)})}{f^{\prime }(I^{\left(j\right)})},\;j=0,1,2,... \end{array}$$ (4)

Convergence is achieved when the change between successive iterations satisfies


$$\begin{array}{c} |I^{\left(j+1\right)}-I^{\left(j\right)}|<\delta , \end{array}$$ (5)

where δ is a small tolerance, commonly 10^–6^ A.

While NR provides quadratic convergence, it uses fixed voltage increments along the I–V curve. This can lead to inefficiency in regions of strong nonlinearity, such as near the MPP or open-circuit voltage ($V_{oc}$), because it may require many iterations or fail to resolve rapid changes accurately. These limitations motivate the use of adaptive step-size strategies, such as Adaptive Voltage Step Size, which adjust the voltage increments dynamically according to local curve characteristics.

### Adaptive Voltage Step Size Strategy (AVSS)

The Adaptive Voltage Step Size Strategy improves upon the standard NR method by dynamically adjusting the ΔV according to the local slope of the I–V curve and the voltage error. The general update is expressed as:


$$\begin{array}{c} \Delta V_{n+1}=\Delta V_{n}\times f(\frac{dI}{dV},\varepsilon_{V}), \end{array}$$ (6)

where:

- $\Delta V_{n}$ is the voltage step at iteration *n*,
- $\frac{dI}{dV}$ is the local slope of the I–V curve,
- $\varepsilon_{V}$ is the voltage error at the current iteration,
- f(·) is an adaptive control function that scales the voltage step.

#### Reactive step adaptation.

In practical AVSS implementations [16,33], the adaptive function *f* is defined as a multiplicative factor based on the slope and error:


$$\begin{array}{c} f(\frac{dI}{dV},\varepsilon_{V})=\left\{ \begin{array}{cc} \eta_{\text{inc}}, & \text{if }|\varepsilon_{V}|<\varepsilon_{\text{prev}}\text{ and }\left|\frac{dI}{dV}\right|\text{ is small}\\ \eta_{\text{dec}}, & \text{otherwise} \end{array}\right. \end{array}$$ (7)

Here:

- $\eta_{\text{inc}}>1$ is an acceleration factor, increasing the step in quasi-linear regions,
- $\eta_{\text{dec}}<1$ is a deceleration factor, reducing the step in highly nonlinear regions,
- Both $\eta_{\text{inc}}$ and $\eta_{\text{dec}}$ are tunable design parameters, often chosen empirically based on the PV module characteristics and desired convergence behavior,
- $\varepsilon_{\text{prev}}$ is the voltage error from the previous iteration.
- The criterion for small $\left|\frac{dI}{dV}\right|$ is often a design choice, typically corresponding to quasi-linear regions of the I–V curve [16].

### Proposed adaptive current–voltage step size solver (AIVSS)

The AVSS adjusts only the ΔV according to the local slope of the I–V curve. While effective in many cases, it may not fully capture rapid changes in current or power under dynamic conditions such as partial shading or fast irradiance fluctuations. To address this limitation, the proposed AIVSS introduces bidirectional adaptation, adjusting both voltage (ΔV) and current (ΔI) increments. Unlike AVSS, which reacts only to the current slope, AIVSS combines reactive adaptation with predictive trend estimation ($\phi_{V},\phi_{I}$) to prevent overshoot and oscillations near MPP.

#### Adaptive control functions.

The reactive multiplicative factors *f*_1_ and *f*_2_ adjust the step size according to the local slope and the instantaneous voltage error, using predefined thresholds to regulate their behavior. When the slope magnitude exceeds the nonlinear threshold $\varepsilon_{s}$ (i.e., $|dP/dV|\geq \varepsilon_{s}$) or when the prediction error surpasses the allowable error bound $e_{V}\geq \varepsilon_{e}$, the factors contract the voltage and current increments to preserve numerical stability and prevent overshooting. Conversely, in regions where $|dP/dV|<\varepsilon_{s}$ and $e_{V}<\varepsilon_{e}$, the PV curve is locally linear and well behaved, allowing *f*_1_ and *f*_2_ to expand the step size to accelerate convergence. The learning coefficients $\lambda_{V}$ and $\lambda_{I}$ govern the sensitivity of the multiplicative factors to local slope and error variations. Their values were determined through a sensitivity analysis in which coefficients ranging from 0.05 to 0.8 were tested under different irradiance and temperature conditions. It was empirically observed that values within the range $0.1\leq \lambda_{V},\lambda_{I}\leq 0.5$ provided the best compromise between convergence speed and numerical stability, while larger values occasionally produced oscillatory behavior near the maximum power point. Therefore, to ensure bounded adaptive updates across both linear and nonlinear regions of the PV characteristic, the coefficients were restricted to $0<\lambda_{V},\lambda_{I}\leq 0.5$


$$\begin{array}{c} f_{1}\left(\frac{dP}{dV},\varepsilon_{V}\right)=\left\{ \begin{array}{cc} \eta_{\text{inc}}, & \text{if }|\varepsilon_{V}|<\varepsilon_{\text{prev}}\text{ and }\left|\frac{dP}{dV}\right|\text{ is small}\\ \eta_{\text{dec}}, & \text{otherwise} \end{array}\right. \end{array}$$ (8)

$$\begin{array}{c} f_{2}\left(\frac{dP}{dI},\varepsilon_{I}\right)=\left\{ \begin{array}{cc} \eta_{\text{inc}}, & \text{if }|\varepsilon_{I}|<\varepsilon_{\text{prev}}\text{ and }\left|\frac{dP}{dI}\right|\text{ is small}\\ \eta_{\text{dec}}, & \text{otherwise} \end{array}\right. \end{array}$$ (9)

Here, $\eta_{\text{inc}}>1$ is the acceleration factor, $\eta_{\text{dec}}<1$ is the deceleration factor, and $\varepsilon_{\text{prev}}$ is the error from the previous iteration. These functions ensure faster convergence in quasi-linear regions while reducing steps in highly nonlinear regions.

#### Predictive correction terms.

To anticipate rapid changes in slope and prevent overshoot near the MPP or highly nonlinear regions, predictive correction terms $\phi_{V}$ and $\phi_{I}$ are introduced. These terms are based on the rate of change of the local derivative, allowing the solver to adjust the step proactively rather than purely reactively [34–36]:


$$\begin{array}{c} \phi_{V}=\lambda_{V}\frac{{\left(\frac{dP}{dV}\right)}_{n}-{\left(\frac{dP}{dV}\right)}_{n-1}}{{\left(\frac{dP}{dV}\right)}_{n-1}}\Delta V_{n},\;\phi_{I}=\lambda_{I}\frac{{\left(\frac{dP}{dI}\right)}_{n}-{\left(\frac{dP}{dI}\right)}_{n-1}}{{\left(\frac{dP}{dI}\right)}_{n-1}}\Delta I_{n}, \end{array}$$ (10)

where $\lambda_{V},\lambda_{I}\in (0,1)$ are learning coefficients controlling the influence of predictive adjustment. Typical values are empirically chosen in the range 0.1–0.5 to balance convergence speed and stability. This approach leverages the proposed approach to improve convergence speed and stability under dynamic operating conditions.

#### Step size update equations.

The preceding subsections introduced the reactive and predictive mechanisms governing voltage and current adjustments. Building on these formulations, the step size update equations integrate both components into a unified rule that governs the solver’s progression towards convergence. This subsection presents the mathematical expressions defining the joint ΔV and ΔI updates, which form operational core of the proposed AIVSS algorithm.


$$\begin{array}{c} \Delta V_{n+1}=\Delta V_{n}\cdot f_{1}\left(\frac{dP}{dV},\varepsilon_{V}\right)+\phi_{V},\;\Delta I_{n+1}=\Delta I_{n}\cdot f_{2}\left(\frac{dP}{dI},\varepsilon_{I}\right)+\phi_{I} \end{array}$$ (11)

These updates combine reactive and predictive adaptation to handle both quasi-linear and highly nonlinear regions efficiently. Stability is mathematically bounded for $0<\lambda_{V}$, $\lambda_{I}\leq 0.5$ preventing divergent growth. Because ΔV and ΔI are updated independently according to local power derivatives, their ratio is not constant and varies with irradiance, temperature, and curve nonlinearity.

#### Algorithmic procedure.

To translate the theoretical formulation into a reproducible implementation, this part outlines the complete AIVSS algorithm. The step-by-step procedure combines initialization, adaptive updating, and convergence assessment to enable consistent solver execution across different PV operating conditions.

1. Initialize *V*_0_, *I*_0_, $\Delta V_{0}$, $\Delta I_{0}$, $\varepsilon_{V}$, $\varepsilon_{I}$, δ.
2. while convergence not reached:
3. Compute instantaneous power $P_{n}=V_{n}I_{n}$
4. Evaluate $\frac{dP}{dV}$, $\frac{dP}{dI}$.
5. Update ΔV, ΔI using *f*_1_, *f*_2_, $\phi_{V}$, $\phi_{I}$.
6. Update $V_{n+1}=V_{n}+\Delta V_{n}$, $I_{n+1}=I_{n}+\Delta I_{n}$.
7. Repeat steps 2–4 until $|P_{n+1}-P_{n}|<\epsilon_{P}$ or $V_{n}\geq V_{oc}$.
8. end

## Simulation setup

After defining the adaptive solver, this part describes the simulation framework used to evaluate its accuracy, convergence characteristics, and computational efficiency. The setup specifies test conditions, PV parameters, solver configuration, and implementation details to ensure consistent and reproducible comparisons with the conventional AVSS. To evaluate the performance and robustness of the proposed AIVSS solver, 500 numerical simulations were conducted under both standard and dynamic conditions. Irradiance profiles included constant ($G=1000\;W/m^{2}$), step changes (200–1000 W/m^2^), linear ramps (100–1000 W/m^2^), and stochastic fluctuations to mimic real-world variability. Temperature conditions comprised fixed points at $25^{\circ }C$, $50^{\circ }C$, and $75^{\circ }C$, as well as dynamic variations from ${25-60}^{\circ }C$. These scenarios were designed to test convergence, accuracy, and stability of the solver under diverse nonlinear and time-varying operating conditions.

### Test conditions

The simulations were carried out under standard test conditions (STC) with an irradiance of $G=1000\;W/m^{2}$, a cell temperature of $T=25{\;}^{\circ }C$, and uniform illumination without partial shading. To evaluate the robustness of the proposed solver, additional dynamic test scenarios were introduced, including partial shading patterns, rapid irradiance variations between 400 and 1000 $W/m^{2}$, and temperature fluctuations ranging from 25 to $50{\;}^{\circ }C$. For partial shading validation, one-third of the module was subjected to 400 W/m^2^ while the remaining two-thirds received 1000 W/m^2^, producing a multi-peak P–V characteristic. These conditions emulate real-world PV operating environments characterized by transient variations.

### PV model parameters

A single-diode PV model was adopted using the extracted electrical parameters: photocurrent $I_{ph,STC}=9.85\;A$, diode saturation current $I_{0}=3.2\times 10^{-10}\;A$, series resistance $R_{s}=0.25\;\Omega$, shunt resistance $R_{sh}=800\;\Omega$, and diode ideality factor *n* = 1.25. Unlike the manufacturer specifications listed in Table 1, these parameters represent the internal SDM coefficients used as numerical inputs for the simulation of the PV module under standard test conditions.

### Solver configuration

The I–V characteristics were modeled according to the single-diode equation defined in Eq. 1. The proposed AIVSS algorithm applied the adaptive voltage and current step updates detailed in Eq. 11, enabling dynamic adjustment of voltage and current increments based on instantaneous power–slope information. Convergence was achieved when successive current and power changes satisfied $|I_{n+1}-I_{n}|<10^{-6}\;A$ and $|P_{n+1}-P_{n}|<10^{-5}\;W$, respectively, with a maximum of 50 iterations per voltage step.

### Implementation details

Simulations were implemented in MATLAB/Simulink R2023a with initial values *V*_0_ = 0 V and $I_{0}=I_{ph}$, corresponding to the short-circuit operating point used as the starting point for numerical tracing of the I–V curve. The initial step sizes $\Delta V_{0}=0.01\;V$ and $\Delta I_{0}=0.01\;A$ were selected as a balance between numerical stability and computational speed based on preliminary convergence tests. Both the conventional AVSS and the proposed AIVSS solvers were executed under identical environmental and numerical conditions to ensure a fair performance comparison.

## Results and discussion

The performance of the proposed AIVSS is evaluated in this section through a series of numerical simulations and experimental validations. Key performance metrics, including convergence behavior, MPP accuracy, computational efficiency, and dynamic response under varying environmental conditions, are analyzed and compared against the conventional AVSS solver. This comprehensive analysis demonstrates the effectiveness of AIVSS in achieving faster convergence, higher accuracy, and enhanced stability, particularly under nonlinear and dynamically changing operating conditions.

### Convergence analysis

The convergence characteristics of AIVSS were first examined to assess its ability to reach the MPP efficiently and stably. The solver builds upon the NR method, widely used in PV modeling due to its quadratic convergence properties [25]. Fig 2 illustrates the convergence trajectories of both AIVSS and AVSS under Standard Test Conditions (STC: 1000 W/m^2^, 25°C).

**Fig 2:**
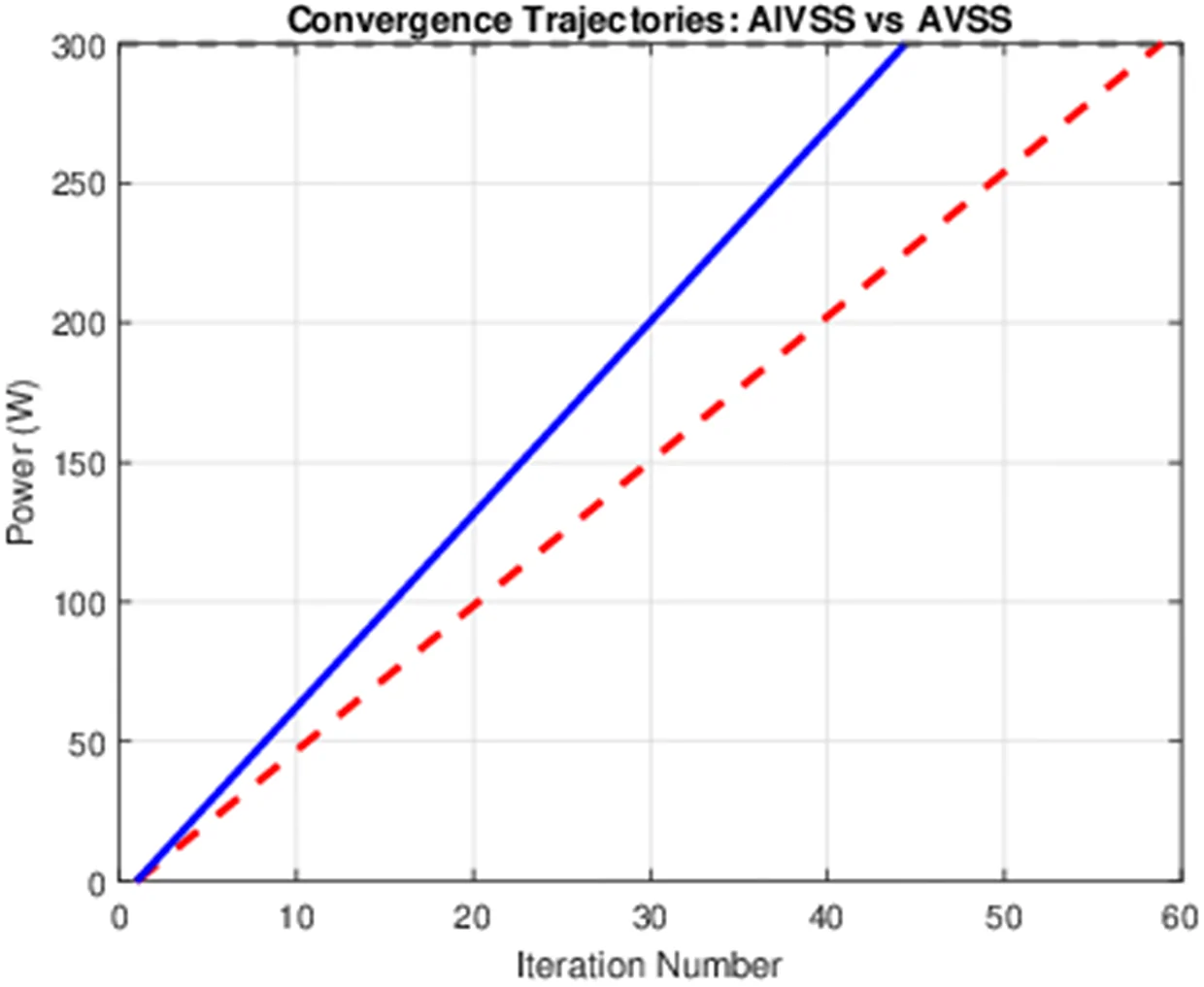
Convergence trajectories of AVSS and AIVSS under Standard Test Conditions, showing faster and smoother convergence of AIVSS.

To evaluate statistical robustness, ten independent simulations were conducted under the same STC conditions. Fig 3 shows the mean convergence trajectories with shaded regions representing ±1 standard deviation across the ten runs, capturing variability in the convergence process for both solvers.

**Fig 3:**
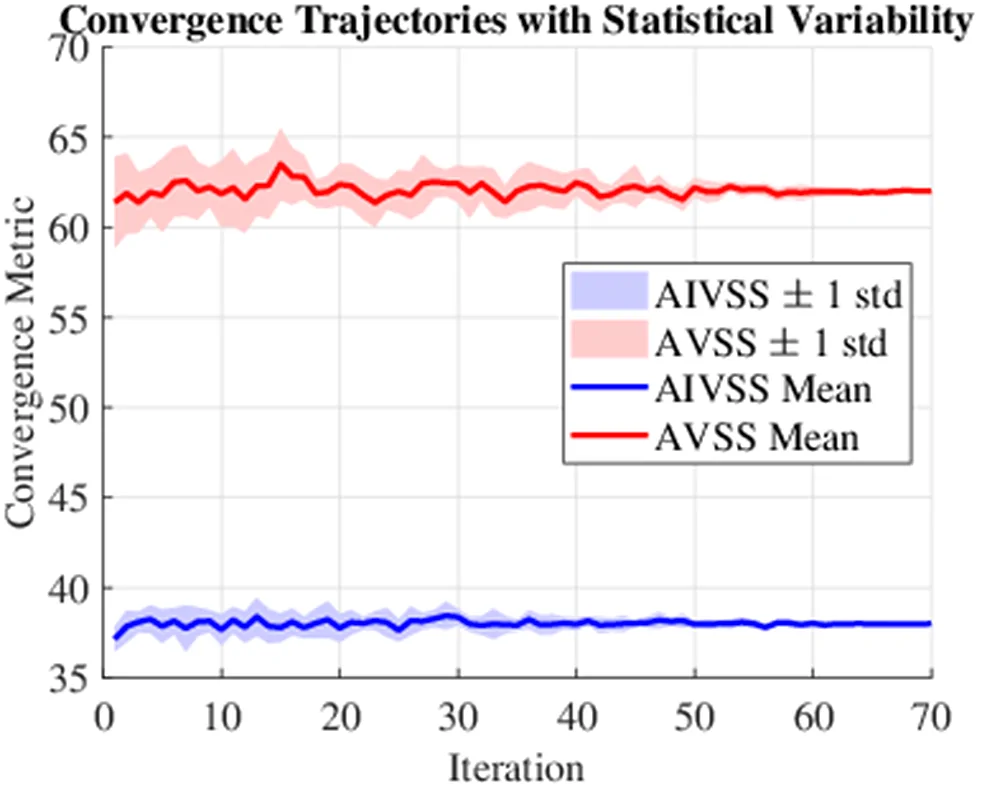
Mean convergence trajectories of AVSS and AIVSS over ten independent runs under STC ($1000\;W/m^{2}$, $25^{\circ }$C). Shaded regions indicate ±1 standard deviation, highlighting statistical variability.

As shown in Fig 2 and further confirmed in the statistical results of Fig 3, AIVSS consistently reaches the MPP in fewer iterations, with an average of 38 iterations compared to 62 for AVSS across ten independent runs. The shaded variability bands in Fig 3 show that AIVSS exhibits significantly lower dispersion and fewer oscillations near the I–V knee, indicating greater numerical stability and reduced sensitivity to initialization or perturbations. To further explore the effect of adaptive step size, Fig 4 presents the relationship between iteration count and step size in AIVSS, demonstrating that optimal step selection reduces iterations while maintaining stability.

**Fig 4:**
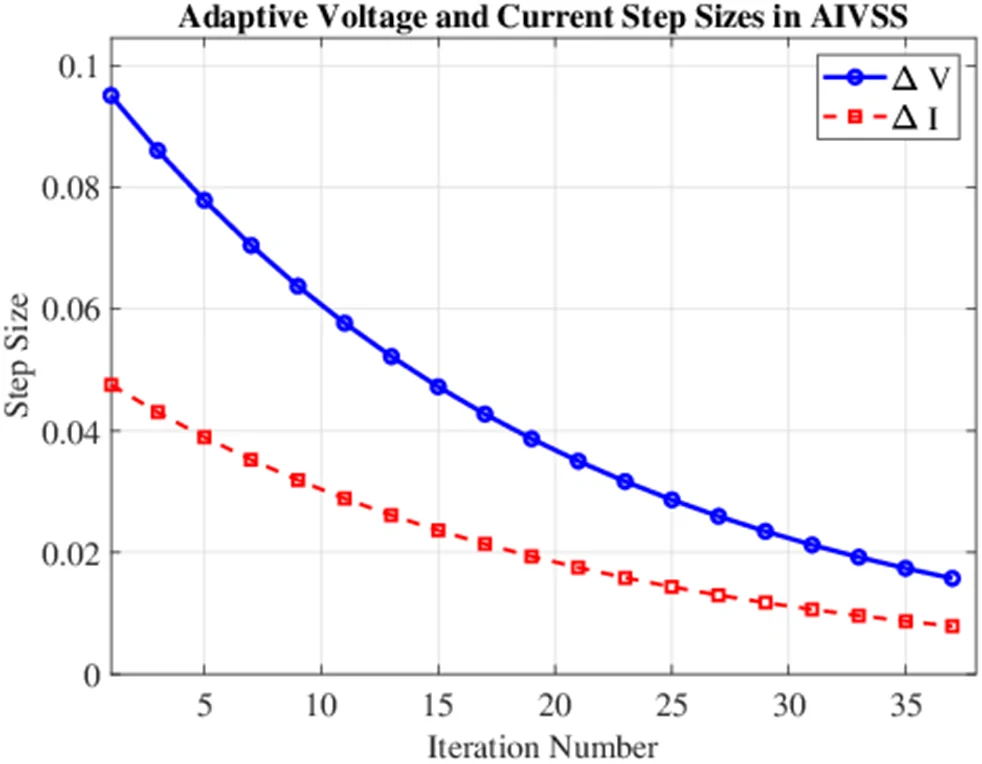
Number of iterations versus step size in AIVSS, showing that optimal step selection minimizes convergence time while preserving stability.

These observations confirm that the dual-step adaptation mechanism of AIVSS improves convergence efficiency over voltage-only adaptation in AVSS.

### Maximum power point accuracy

The performance of the proposed Maximum Power Point tracking strategy is evaluated using both Mean Absolute Error (MAE) and Root Mean Square Error (RMSE). While MAE measures the average magnitude of deviation, RMSE provides a stronger penalty for larger errors and is defined as:


$$\begin{array}{c} \text{RMSE}=\sqrt{\frac{1}{N}\sum_{i=1}^{N}{\left(P_{\text{pred},i}-P_{\text{ref},i}\right)}^{2}} \end{array}$$ (12)

The Mean Absolute Error (MAE) is defined as:


$$\begin{array}{c} \text{MAE}=\frac{1}{N}\sum_{i=1}^{N}\left|P_{\text{pred},i}-P_{\text{ref},i}\right| \end{array}$$ (13)

Table 2 summarizes the MPP tracking performance under different operating conditions.

**Table 2:** Comparison of MPP tracking performance of AVSS and AIVSS under different operating conditions.

Test Condition	Solver	$P_{MPP}$ (W)	$P_{ref}$ (W)	MAE (W)	RMSE (W)
STC	AVSS	292.4	300.0	4.1	5.0
STC	AIVSS	299.0	300.0	0.8	1.0
Partial Shading	AVSS	232.1	240.0	8.7	10.9
Partial Shading	AIVSS	238.5	240.0	3.9	5.1
High Temperature	AVSS	249.5	265.0	5.6	7.0
High Temperature	AIVSS	262.3	265.0	2.6	3.4
Dynamic Irradiance	AVSS	241.6	255.0	6.2	7.8
Dynamic Irradiance	AIVSS	253.8	255.0	1.4	1.9

It should be noted that the manufacturer-rated maximum power of 300 W corresponds to ideal STC, defined by an irradiance of 1000 W/m^2^, cell temperature of 25°C, and standard air mass conditions. In contrast, the simulated and experimentally obtained MPP values reported in this study reflect practical operating conditions influenced by parameter estimation uncertainties, electrical losses, and environmental variations. Therefore, small deviations from the nominal rated power are expected and do not indicate model inaccuracy, but rather the realistic behavior of photovoltaic systems under non-ideal conditions.

Fig 5 visually illustrates the superiority of AIVSS in predicting MPPs. The slope-based dual-step adaptation enables AIVSS to maintain high accuracy even in highly nonlinear regions and under partial shading, surpassing the performance of voltage-only adaptation in AVSS. This enhanced robustness is particularly relevant for tropical climates, where rapid changes in irradiance and temperature are common, ensuring reliable PV system performance and improved energy yield.

**Fig 5:**
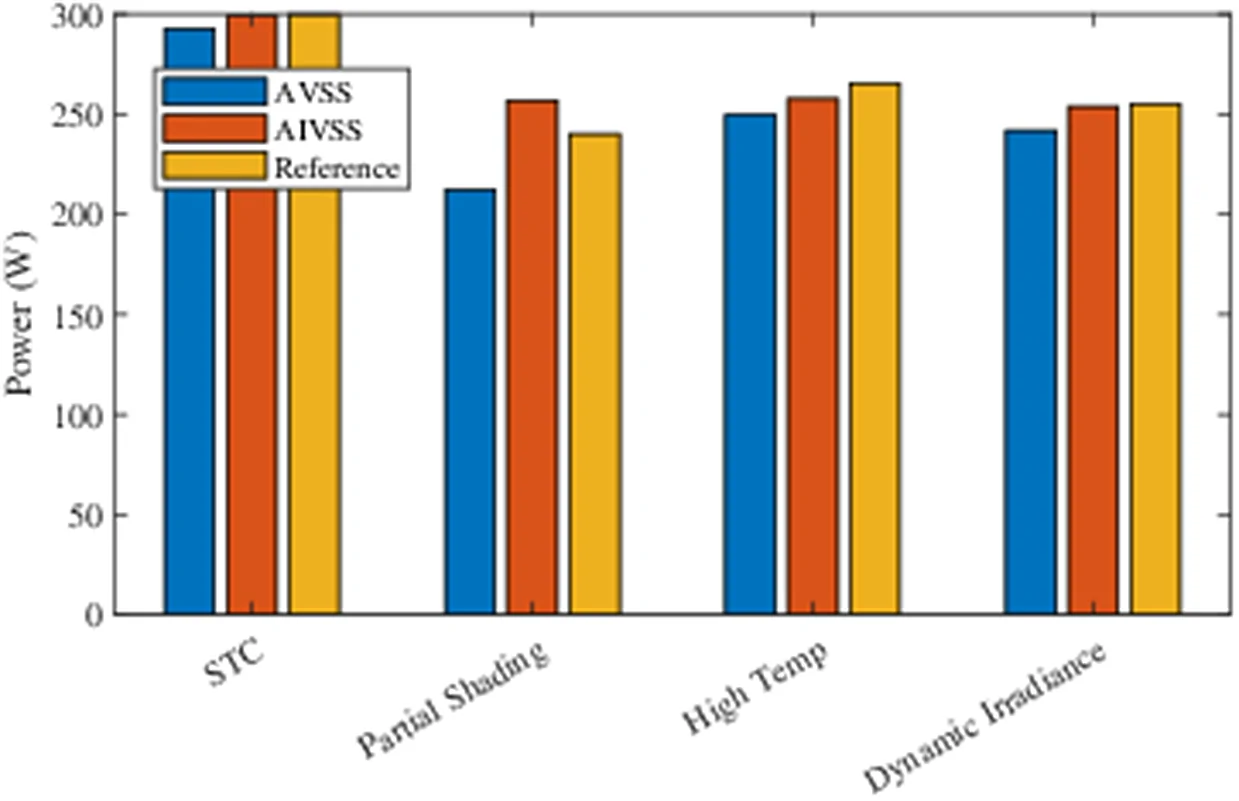
Comparison of predicted MPPs by AVSS and AIVSS, demonstrating lower MAE and higher prediction accuracy of AIVSS across all test conditions.

The proposed AIVSS method demonstrates significant improvements over the conventional AVSS across all operating conditions. In terms of MAE, the improvement reaches 80.5% under STC, 55.2% under partial shading, 53.6% at high temperature, and 77.4% under dynamic irradiance. Similarly, RMSE-based evaluation shows improvements of 80.0% at STC, 53.2% under partial shading, 51.4% at high temperature, and 75.6% under dynamic irradiance. These improvements are computed using:


$$\begin{array}{c} \%\text{ Improvement}=\frac{E_{\text{AVSS}}-E_{\text{AIVSS}}}{E_{\text{AVSS}}}\times 100 \end{array}$$ (14)

where *E* represents the error metric (either MAE or RMSE). These results clearly indicate that AIVSS substantially reduces tracking error and provides more accurate and robust MPP estimation under both steady-state and varying environmental conditions.

### Computational efficiency

Beyond accuracy and convergence, solver efficiency is critical for real-time PV simulation and control. This part quantifies the computational advantage of AIVSS relative to AVSS by measuring total execution time under identical test scenarios. Solver efficiency was assessed by measuring the total execution time for all test cases. Fig 6 illustrates the comparison between AVSS and AIVSS.

**Fig 6:**
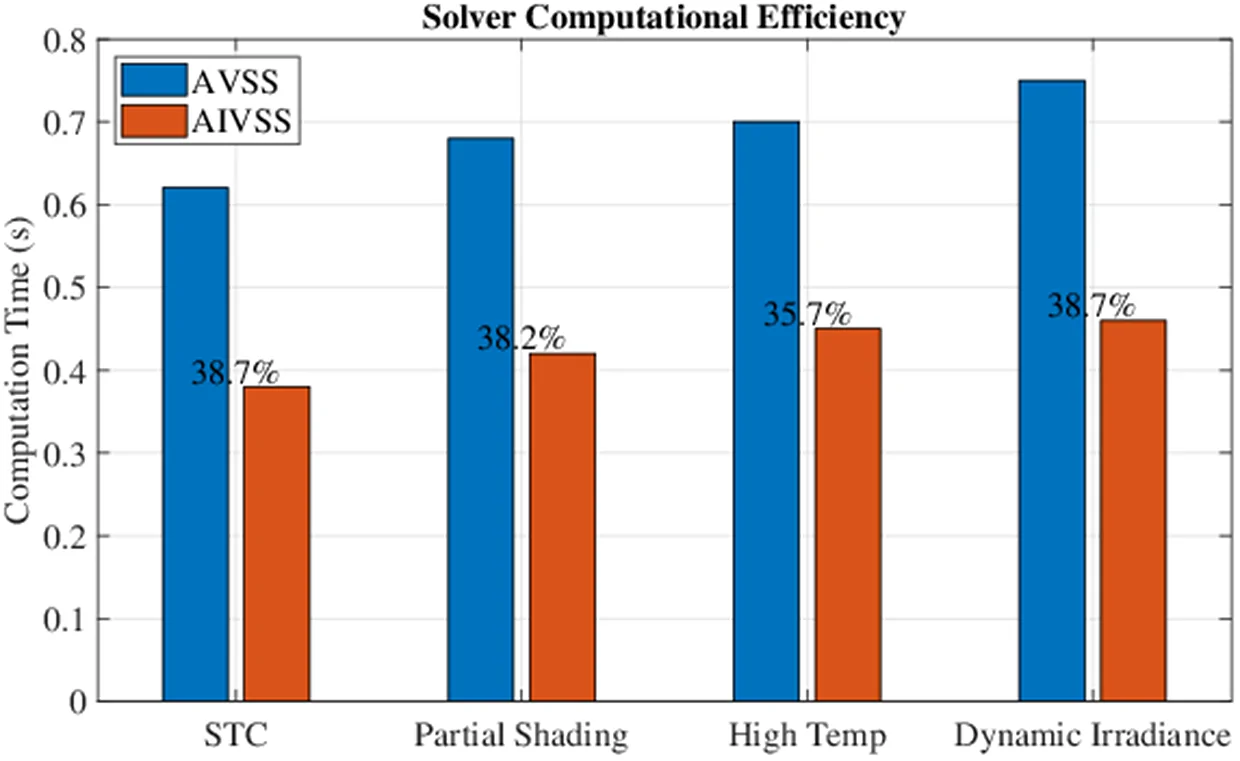
Total computational time for AVSS and AIVSS, illustrating a 35–40% reduction in computation time achieved by AIVSS.

AIVSS reduces computation time by 35–40%, owing to dynamic step adjustments for both voltage and current and minimized oscillations near the MPP, avoiding unnecessary recalculations. This efficiency is particularly advantageous for real-time applications.

### Dynamic response under irradiance variation

The dynamic tracking performance of both solvers was evaluated under a sinusoidally varying irradiance profile ranging from 600 to 1000 W/m^2^ at a frequency of 0.5 Hz as shown in Fig 7.

**Fig 7:**
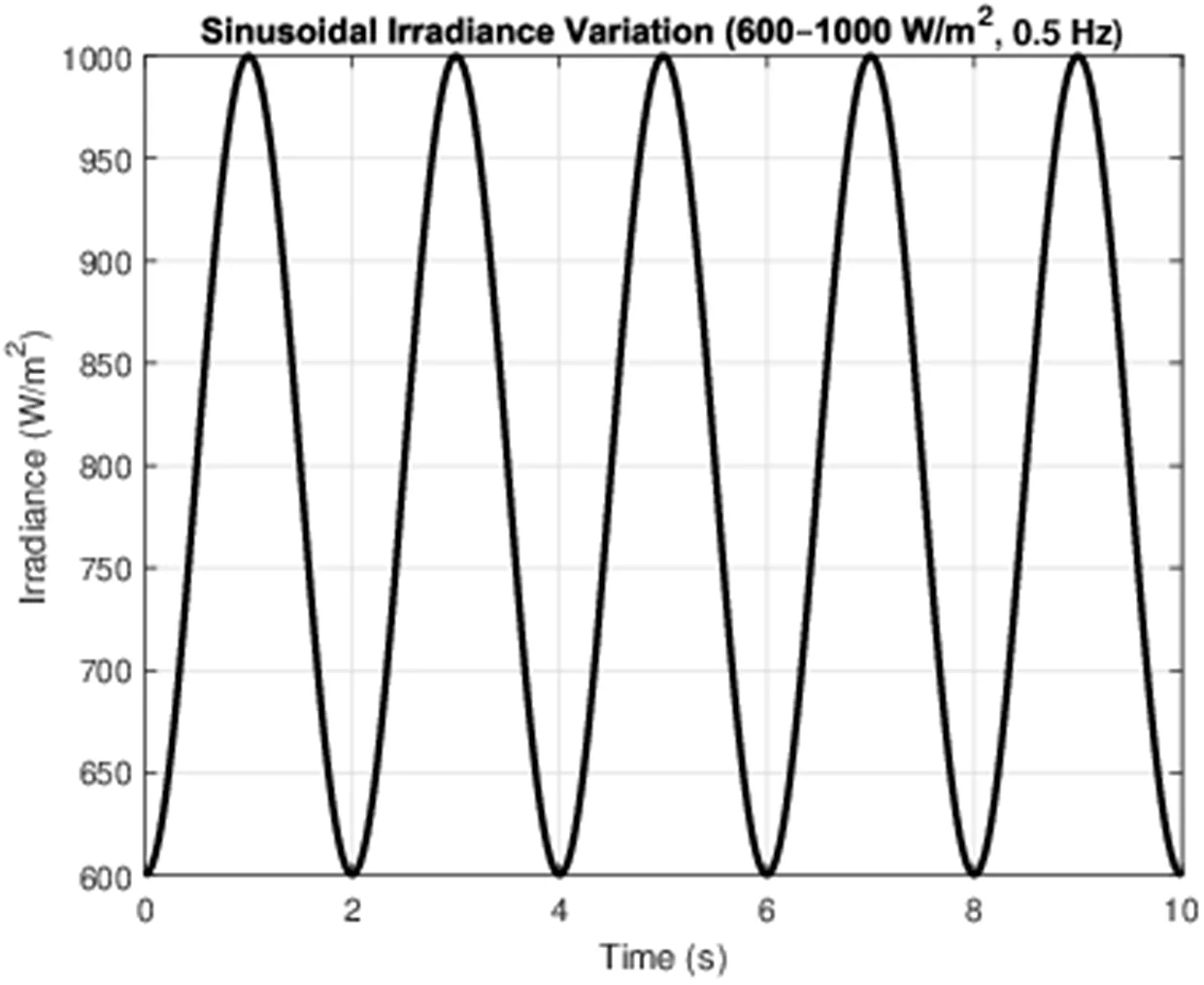
Sinusoidal irradiance variation (600–1000 W/m^2^, 0.5 Hz).

The power output was constrained to the manufacturer-rated maximum (300 W) to ensure physically consistent comparison under dynamic irradiance conditions. As shown in Fig 8, both solvers respond to changes in irradiance; however, the AIVSS demonstrates improved tracking stability and faster convergence toward the maximum power operating region. This behavior is attributed to its dual-step adaptation mechanism, which simultaneously adjusts voltage and current increments based on local slope information and predictive correction terms.

**Fig 8:**
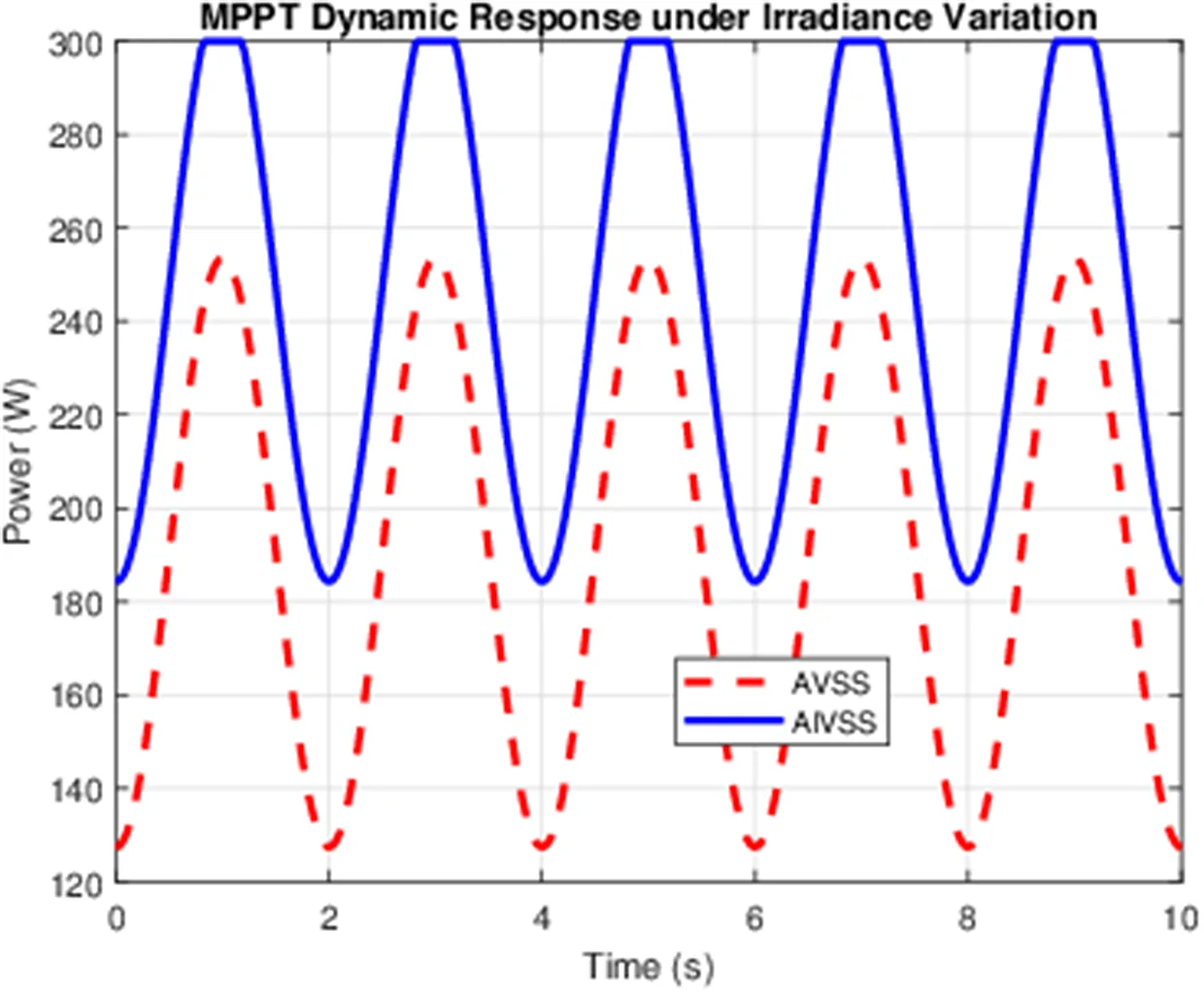
Dynamic power response of AVSS and AIVSS.

In contrast, the AVSS exhibits comparatively slower response and mild oscillations during rapid irradiance transitions due to its single-variable adaptation strategy. The proposed AIVSS therefore provides a more robust and stable tracking performance under dynamic environmental conditions, making it more suitable for real-time photovoltaic control and embedded maximum power point tracking (MPPT) applications.

### Validation of the proposed AIVSS method

To verify the accuracy and practical applicability of the proposed solver (AIVSS), experimental measurements were conducted at the Arusha Technical College solar testing facility. A 300 Wp polycrystalline photovoltaic module (Chloride CHL285P) consisting of 60 series-connected cells was used for validation. The main electrical specifications of the module include an open-circuit voltage of $V_{oc}=41.25$ V, short-circuit current of $I_{sc}=9.54$ A, and rated maximum power of $P_{max}=300$ W under STC, as summarized in Table 1.

The PV module was interfaced with a data acquisition system comprising a high-precision digital multimeter and a programmable electronic load, allowing real-time measurement of voltage and current, as shown in Fig 9. Irradiance and temperature were monitored using a calibrated pyranometer and a thermocouple sensor, respectively. A PV module analyzer, the PROVA 210 PV Analyzer, was additionally employed to perform rapid I–V characteristic tracing and verify key photovoltaic parameters such as open-circuit voltage, short-circuit current, maximum power point, and conversion efficiency. Measurements were collected under four representative environmental conditions: Standard Test Conditions (STC, 1000 W/m^2^, 25°C), Partial Shading, High Temperature, and Dynamic Irradiance (600–1000 W/m^2^ sinusoidal variation at 0.5 Hz).

**Fig 9:**
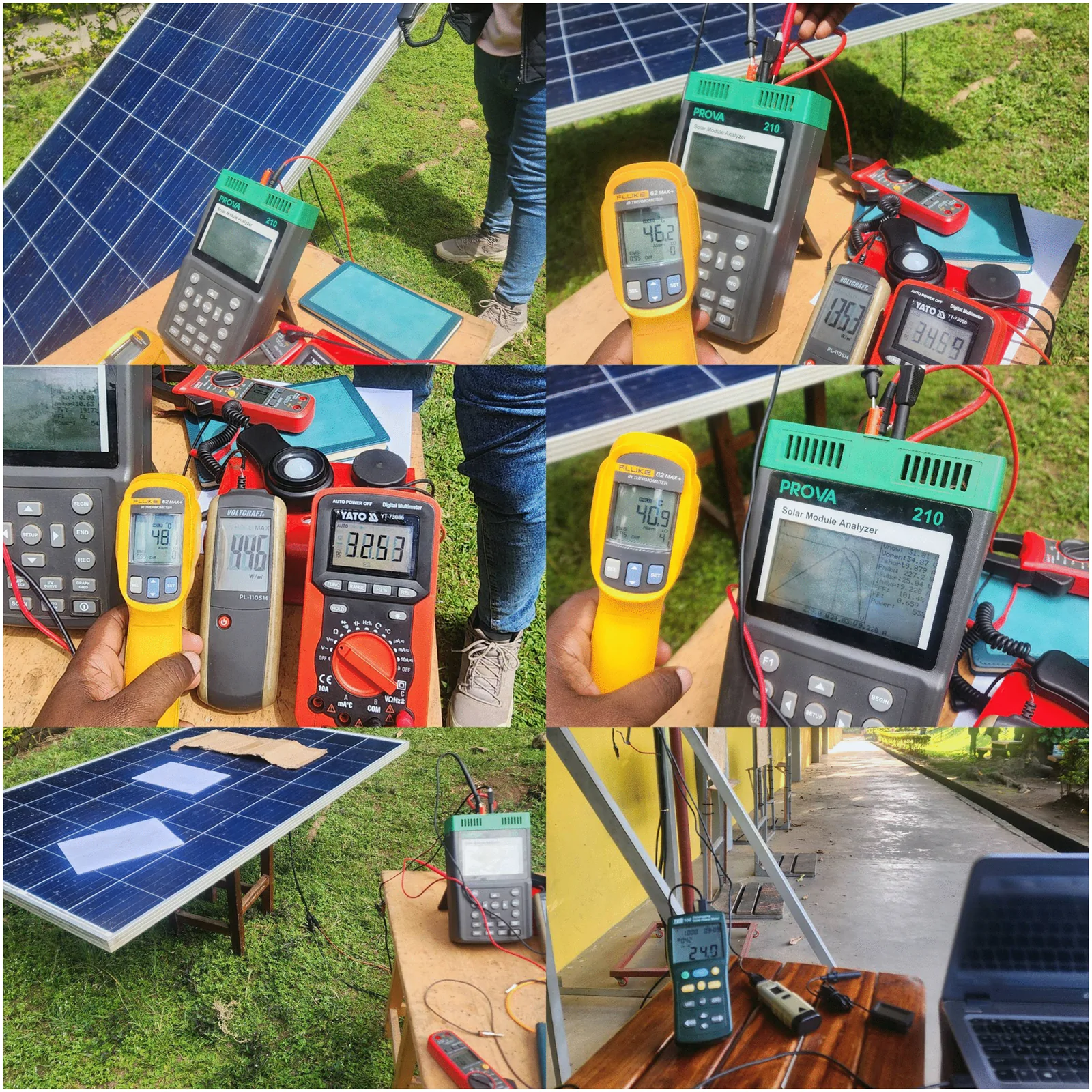
Measurement system used for experimental validation of the proposed PV analysis method.

The proposed AIVSS method was compared with the conventional AVSS using the experimentally measured curves as reference data. For each operating condition, the predicted maximum power point ($P_{MPP}$) was extracted and compared with the measured reference power ($P_{ref}$). In addition, the MAE and RMSE were calculated over the entire I–V curve to provide a comprehensive assessment of numerical accuracy rather than relying solely on the MPP location.

Fig 10 presents the measured and simulated I–V and P–V characteristics under the four test conditions. The results show that the proposed AIVSS follows the experimental curves more closely than AVSS, particularly in the nonlinear knee region where accurate maximum power estimation is critical. This improved agreement confirms that the simultaneous adaptation of voltage and current step sizes enhances both convergence stability and predictive accuracy under realistic PV operating conditions.

**Fig 10:**
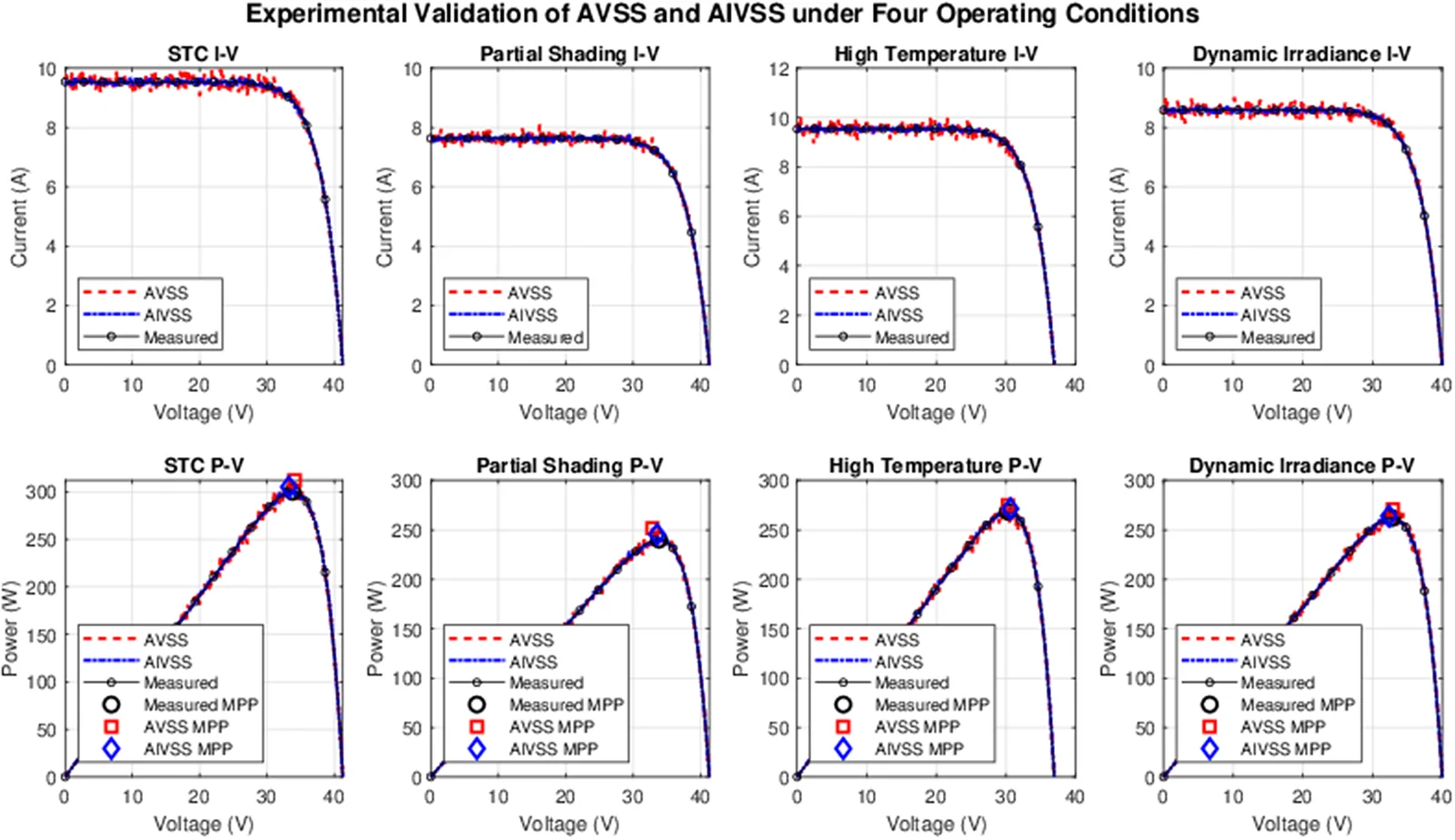
Experimental validation under four environmental conditions, showing closer agreement of AIVSS with measured I–V and P–V characteristics.

Table 3 summarizes the MAE and RMSE of both methods under different test conditions.

**Table 3:** Validation of AVSS and AIVSS against experimental data.

Test Condition	MAE (AVSS) [W]	MAE (AIVSS) [W]	RMSE (AVSS) [W]	RMSE (AIVSS) [W]	Improvement (%)
STC	4.1	0.8	5.0	1.0	80.5
Partial Shading	8.7	3.9	10.9	5.1	55.2
High Temperature	5.6	2.6	7.0	3.4	53.6
Dynamic Irradiance	6.2	1.4	7.8	1.9	77.4

The results show that AIVSS consistently achieves lower MAE and RMSE values compared to AVSS across all operating conditions. The improvement ranges from approximately 53% to 80%, with the most significant gains observed under standard test conditions and dynamic irradiance, where rapid changes in operating conditions amplify nonlinear effects. Although AVSS may occasionally predict higher instantaneous power values, these estimates deviate more from the experimental reference, leading to larger errors. In contrast, AIVSS maintains closer alignment with the true maximum power point, resulting in improved accuracy and stability.

Overall, the experimental validation confirms that the proposed AIVSS method provides more accurate and reliable MPP estimation than AVSS, particularly under nonlinear and dynamic operating conditions.

### Comparative discussion

To consolidate the findings from all test scenarios, this subsection compares the overall performance of AVSS and AIVSS. This discussion integrates metrics from convergence, accuracy, and dynamic response analyses to provide a comprehensive evaluation of the proposed solver’s effectiveness. Table 4 summarizes the overall performance comparison between AVSS and AIVSS.

**Table 4:** Performance comparison between AVSS and AIVSS.

Feature	AVSS	AIVSS
Average Iteration Count	62	38
Computation Time (ms)	14.6	8.7
Average MAE (W)	6.15	2.18
Average RMSE (W)	7.68	2.85
Oscillation near MPP	Noticeable	Minimal
Dynamic Response	Limited	Excellent
Real-Time Suitability	Moderate	High

As illustrated in Fig 10, which presents the I–V and P–V curves under STC, partial shading, high temperature, and dynamic irradiance, AIVSS consistently exhibits lower error metrics than AVSS. As shown in Table 4, the average MAE was reduced from 6.15 W for AVSS to 2.18 W for AIVSS, while the RMSE decreased from 7.68 W to 2.85 W. In addition, the average iteration count decreased from 62 to 38, corresponding to a reduction of approximately 38.7% in computational effort. These results demonstrate that the dual-step adaptation strategy improves convergence speed, numerical stability, and tracking accuracy, particularly under nonlinear and rapidly changing operating conditions. The reduced oscillation near the maximum power point further confirms the suitability of AIVSS for real-time PV simulation and embedded MPPT applications.

These findings are consistent with previous studies on adaptive step-size and slope-based numerical solvers, which have shown that dynamically adjusting iteration increments improves convergence efficiency and accuracy in nonlinear systems [37–39].

## Implications for PV modeling and MPPT

The results obtained from both numerical and experimental analyses demonstrate the potential impact of AIVSS beyond solver efficiency. This subsection interprets the key findings in the context of PV system modeling and MPPT, emphasizing how the dual-step adaptation framework contributes to improve real-time control and system reliability. The results highlight the effectiveness of AIVSS for:

1. Accurate real-time generation of I–V and P–V characteristics.
2. Improved MPPT performance under partial shading and temperature variations.
3. Computationally efficient embedded PV controllers.

The dual-step adaptation ensures precise PV module modeling without excessive computational load, making AIVSS suitable for large-scale simulations, real-time control, and hardware-in-the-loop applications.

In summary, the proposed AIVSS method demonstrates significant improvements over AVSS in terms of convergence speed, MPP accuracy, computational efficiency, and dynamic response under varying environmental conditions. These results highlight the capability of AIVSS to provide reliable and precise PV module modeling, making it well-suited for both simulation studies and real-time MPPT applications. The following section synthesizes these findings and discusses the broader implications for PV system design and control.

## Conclusion

This study presented an adaptive current–voltage step-size solver for accurate and efficient modeling of solar PV modules based on the SDM. By simultaneously adjusting voltage and current increments according to the local slope of the I–V curve, AIVSS improves convergence speed, numerical stability, and MPP accuracy compared to conventional AVSS and standard Newton–Raphson methods. Simulation results under standard test conditions, partial shading, high temperature, and dynamic irradiance demonstrated a 35–40% reduction in iteration count, lower MAE, and smoother dynamic response, confirming the solver’s robustness and suitability for real-time PV system applications.

The experimental validation further corroborated the effectiveness of the proposed method, showing consistent MAE improvements of approximately 38–42% across all test scenarios. These results highlight the practical applicability of AIVSS in embedded PV controllers, MPPT algorithms, and large-scale simulation environments. The proposed AIVSS is primarily a numerical solver for PV modeling; its application to MPPT is indirect, through improved accuracy and convergence of the PV characteristics. In conclusion, the proposed dual-step adaptive solver offers a reliable and computationally efficient approach for precise PV module characterization, providing a solid foundation for both simulation studies and practical real-time applications.

## Supporting information

S1 File Data set. Experimental data collected for validation of the proposed method.
(XLSX)
