# Supplementary material for: An adaptive current–voltage step size solver for accurate and efficient modeling of solar photovoltaic modules
Source: PLoS One. 2026 Oct 6;21(10):e0359796. doi: 10.1371/journal.pone.0359796 (PMC13641713; doi:10.1371/journal.pone.0359796)
Supplement: Supplementary file 1 [file pone.0359796.s003.pdf]

Dear Editors and Reviewers,

We sincerely thank you for your thorough evaluation and constructive feedback. Your comments have significantly helped improve the quality, clarity, and technical rigor of the manuscript. We have carefully revised the paper accordingly, and our detailed responses are provided below.

1. Academic Editor Comments

a) PLOS ONE Style Requirements

We carefully reviewed the manuscript and updated it to comply with the PLOS ONE formatting and style requirements, including the appropriate file naming conventions and formatting guidelines provided in the official PLOS templates.

b) Code Sharing Requirements

We reviewed the PLOS ONE policy regarding code sharing and reproducibility. The author-generated code underlying the findings of this study has been organized and prepared for public availability in accordance with the journal guidelines.

c) Use of the PLOS LaTeX Template

The manuscript has been fully updated using the official PLOS LaTeX template as requested. Formatting, bibliography style, figures, tables, and section structures were revised to ensure consistency with PLOS submission requirements.

d) Supporting Information Captions

Captions for all Supporting Information files have now been included at the end of the manuscript. In-text citations referring to the Supporting Information files were also revised to ensure consistency with the captions and journal guidelines.

e) Reviewer-Suggested References

We carefully evaluated all reviewer-recommended references. Relevant publications have been incorporated into the revised manuscript where appropriate. References deemed not directly relevant to the scope or methodology of the present work were not included.

2. Reviewer #1

Comment 1

The abstract should provide the main outcomes of the study.

Response

Thank you for this suggestion. The abstract has been revised to explicitly include the key quantitative outcomes of the study. Specifically, we now report the reduction in iteration count (35–40%), improvements in MAE and RMSE, and computational efficiency gains to better reflect the main findings.

Revision made: Abstract section

Comment 2

The novelties and contributions of the study should be clearly stated at the end of the Introduction section.

Response

We agree with this comment. A new paragraph has been added at the end of the Introduction clearly outlining the main contributions of the paper, including:

1. Development of the dual adaptive current–voltage step-size solver (AIVSS),
2. Integration of predictive correction terms,
3. Improved convergence and stability under nonlinear operating conditions, and
4. Experimental validation under real operating conditions.

Revision made: End of the Introduction section

Comment 3

Abbreviations should be defined only once.

Response

This issue has been corrected throughout the manuscript. All abbreviations are now defined at their first occurrence and used consistently thereafter without redefinition (e.g., PV, SDM, AVSS, and AIVSS).

Revision made: Throughout the manuscript

Comment 4

Under STC, the real MPP is 300 W, which differs from Table 2.

Response

The reviewer correctly noted the discrepancy between the rated module power (300 W) and the simulated MPP values reported in Table 2. The original simulations were performed using a single-cell equivalent parameter set rather than the full module-scaled parameter set, resulting in lower predicted absolute power values. In the revised manuscript, the SDM parameters have been rescaled to represent the full 60-cell module so that the simulated MPP under STC more closely matches the manufacturer-rated value. Accordingly, the revised manuscript now includes the reference (rated) MPP values in Table 2 and presents performance evaluation using both MAE and RMSE for a more rigorous comparison.

Revision made: Section 4.1.2 (PV Model Parameters), Table 2, and Results Discussion

Comment 5

AVSS shows higher predicted power but higher MAE than AIVSS.

Response

We appreciate this observation. The higher predicted power obtained using AVSS does not necessarily indicate higher accuracy. In several cases, AVSS slightly overestimates the MPP, leading to larger deviations from the reference values. In contrast, AIVSS provides predictions that are closer to the true/reference MPP, resulting in lower MAE and RMSE values.

This explanation has been clarified in the revised manuscript.

Revision made: Section 5.2 (Performance Evaluation and Error Analysis)

Comment 6

Fig. 7 should be split.

Response

This suggestion has been implemented. Figure 7 has been divided into two separate figures:

- One illustrating irradiance variation, and
- Another showing the power tracking response.

This modification improves clarity and readability.
Revision made: Figs 7 and 8

Comment 7

Provide system parameters and experimental setup photos.

Response

Additional details of the experimental setup have been included in the revised manuscript, including:

- Measurement instruments and their specifications,
- System parameters, and
- Sensor descriptions.

Furthermore, photographs of the experimental setup have been added to enhance clarity and reproducibility.
Revision made: Section 4.1.1 (Experimental Setup and Test Conditions) and Fig. 1

Comment 8

Mismatch between STC (300 W) and Fig. 8 results.

Response

This issue is related to the explanation provided in Comment 4. The observed difference arises from the original modeling assumptions and operating conditions. The manuscript has been revised to clearly explain this distinction and avoid confusion.
Revision made: Section 5.3 (Experimental Validation Results)

Comment 9

Inconsistency between Fig. 8 and Table 3.

Response

We acknowledge this inconsistency. Table 3 has been revised to ensure consistency with the experimental results presented in Fig. 10. Additionally, a more detailed discussion has been included to explain the observed differences between AVSS and AIVSS performance.
Revision made: Table 3 and Section 5.3 (Experimental Discussion)

Comment 10

MAE alone is insufficient.

Response

We agree with this valuable suggestion. Additional performance metrics have now been incorporated, including:

- Root Mean Square Error (RMSE),
- Convergence time, and
- Iteration count.

These metrics provide a more comprehensive evaluation of solver performance.
Revision made: Abstract, Section 5.2, Tables 2–4

Comment 11

Discussion should be more rigorous.

Response

The Results and Discussion section has been substantially expanded. The revised manuscript now includes:

- Quantitative comparisons,
- Deeper analysis of convergence behavior,
- Detailed interpretation of experimental results, and
- Improved linkage between figures, tables, and conclusions.

Revision made: Results and Discussion section

3. Reviewer #2

Comment 1

How were the adaptation conditions for λ_V and λ_I determined?

Response

Thank you for this important observation. We have clarified in the manuscript that the learning coefficients λ_V and λ_I were selected through sensitivity analysis under varying irradiance and temperature conditions. The revised text now explains that values in the range of 0.1 to 0.5 provided the best compromise between convergence speed and numerical stability, while larger values produced oscillatory behavior near the maximum power point.
Revision made: Section 3.3.1 (Adaptive Control Functions)

Comment 2

The temperature coefficient of voltage (β) is not considered, even though temperature varies. How does this omission affect the MPP accuracy?

Response

Thank you for this valuable comment. We have clarified that the voltage temperature coefficient (β) was not explicitly incorporated because the primary objective of this study is to evaluate the numerical performance of the proposed AIVSS solver rather than to develop a detailed temperature-dependent parameter extraction model. Since both AVSS and AIVSS were evaluated under identical modeling assumptions, the comparative assessment of the proposed method remains valid.
Revision made: Section 2.2 (Parameter Dependence)

Comment 3

What is the purpose of the panel data presented in Section 3.5.2? Please clarify whether it is used in the analysis.

Response

Thank you for pointing this out. We have clarified that the manufacturer module specifications presented in Table 1 serve as reference data for the experimental PV module, while the parameters listed in the "PV Model Parameters" subsection correspond to the extracted single-diode model coefficients used as numerical inputs in the simulations.

Revision made: Section 4.1.2 (PV Model Parameters)

Comment 4

Will the step size ratio of voltage and current remain constant under all irradiance conditions?

Response

Thank you for this question. We have clarified that the voltage and current step sizes are updated independently according to local power derivatives. Therefore, the ratio $\Delta V/\Delta I$ does not remain constant and varies with irradiance, temperature, and local nonlinearity of the PV characteristic.

Revision made: Section 3.3.3 (Step Size Update Equations)

Comment 5

What shading patterns and high-temperature values were used under different test conditions? Please specify them clearly.

Response

Thank you for this suggestion. We have now explicitly described the partial shading condition used in the validation, where one-third of the module was subjected to 400 W/m^2 while the remaining two-thirds received 1000 W/m^2. The temperature conditions were also clarified as 25°C, 50°C, and 75°C for the high-temperature simulations.

Revision made: Section 4.1.1 (Test Conditions)

Comment 6

RMSE analysis is not included in the paper. Please add it to validate the accuracy of the proposed method.

Response

Thank you for this suggestion. RMSE analysis has now been incorporated throughout the manuscript, including the methodology, performance tables, and discussion section, to provide additional validation of the proposed method's accuracy.

Revision made: Abstract, Section 5.2, Tables 2 and 4

Comment 7

It is not clearly stated whether the proposed method contributes to PV modeling or MPPT tracking. Please clearly distinguish between these two aspects.

Response

Thank you for this observation. We have revised the Introduction to clarify that the primary contribution of this work is the improvement of numerical PV module modeling through adaptive dual-step solving, while its application to MPPT is considered a secondary implementation benefit.

Revision made: Introduction section

We sincerely appreciate the insightful comments and valuable suggestions provided by the editors and reviewers. Their feedback has significantly improved the clarity, technical depth, and overall quality of the manuscript.

Sincerely,
Nsulwa John Mlazi
