# Supplementary material for: An adaptive current–voltage step size solver for accurate and efficient modeling of solar photovoltaic modules
Source: PLoS One. 2026 Oct 6;21(10):e0359796. doi: 10.1371/journal.pone.0359796 (PMC13641713; doi:10.1371/journal.pone.0359796)
Supplement: Peer review history

# Peer review: Decision Letter 0

**DOI:** 10.1371/journal.pone.0359796.r001

**Authors:** Shafaat Ullah

7 Apr 2026

Dear Dr. Mlazi,

Thank you for submitting your manuscript to PLOS ONE. After careful consideration, we feel that it has merit but does not fully meet PLOS ONE’s publication criteria as it currently stands. Therefore, we invite you to submit a revised version of the manuscript that addresses the points raised during the review process.

==============================

The manuscript requires major revisions before it can be considered for publication. Significant improvements are needed in the technical depth, clarity of presentation, and adequacy of supporting results. The authors are requested to carefully address all reviewer comments and substantially revise the manuscript.

==============================

Please submit your revised manuscript by May 22 2026 11:59PM. If you will need more time than this to complete your revisions, please reply to this message or contact the journal office at plosone@plos.org. When you're ready to submit your revision, log on to https://www.editorialmanager.com/pone/ and select the 'Submissions Needing Revision' folder to locate your manuscript file.

- A letter that responds to each point raised by the academic editor and reviewer(s). You should upload this letter as a separate file labeled 'Response to Reviewers'.
- A marked-up copy of your manuscript that highlights changes made to the original version. You should upload this as a separate file labeled 'Revised Manuscript with Track Changes'.
- An unmarked version of your revised paper without tracked changes. You should upload this as a separate file labeled 'Manuscript'.

If you would like to make changes to your financial disclosure, please include your updated statement in your cover letter. Guidelines for resubmitting your figure files are available below the reviewer comments at the end of this letter.

If applicable, we recommend that you deposit your laboratory protocols in protocols.io to enhance the reproducibility of your results. Protocols.io assigns your protocol its own identifier (DOI) so that it can be cited independently in the future. For instructions see: https://journals.plos.org/plosone/s/submission-guidelines#loc-laboratory-protocols. Additionally, PLOS ONE offers an option for publishing peer-reviewed Lab Protocol articles, which describe protocols hosted on protocols.io. Read more information on sharing protocols at https://plos.org/protocols?utm_medium=editorial-email&utm_source=authorletters&utm_campaign=protocols.

As the corresponding author, your ORCID iD is verified in the submission system and will appear in the published article. PLOS supports the use of ORCID, and we encourage all coauthors to register for an ORCID iD and use it as well. Please encourage your coauthors to verify their ORCID iD within the submission system before final acceptance, as unverified ORCID iDs will not appear in the published article. *Only*  the individual author can complete the verification step; PLOS staff *cannot*  verify ORCID iDs on behalf of authors.

We look forward to receiving your revised manuscript.

Kind regards,

Shafaat Ullah, PhD in Electrical Engineering

Academic Editor

PLOS One

Journal Requirements:

When submitting your revision, we need you to address these additional requirements.

1. Please ensure that your manuscript meets PLOS ONE's style requirements, including those for file naming. The PLOS ONE style templates can be found at

https://journals.plos.org/plosone/s/file?id=wjVg/PLOSOne_formatting_sample_main_body.pdf and

https://journals.plos.org/plosone/s/file?id=ba62/PLOSOne_formatting_sample_title_authors_affiliations.pdf

2. Please note that PLOS One has specific guidelines on code sharing for submissions in which author-generated code underpins the findings in the manuscript. In these cases, we expect all author-generated code to be made available without restrictions upon publication of the work. Please review our guidelines at https://journals.plos.org/plosone/s/materials-and-software-sharing#loc-sharing-code and ensure that your code is shared in a way that follows best practice and facilitates reproducibility and reuse.

3. Please update your submission to use the PLOS LaTeX template. The template and more information on our requirements for LaTeX submissions can be found at http://journals.plos.org/plosone/s/latex.

4. Please include captions for your Supporting Information files at the end of your manuscript, and update any in-text citations to match accordingly. Please see our Supporting Information guidelines for more information: http://journals.plos.org/plosone/s/supporting-information.

5. If the reviewer comments include a recommendation to cite specific previously published works, please review and evaluate these publications to determine whether they are relevant and should be cited. There is no requirement to cite these works unless the editor has indicated otherwise.

**Additional Editor Comments:**

Dear authors,

The manuscript requires major revisions before it can be considered for publication. Significant improvements are needed in the technical depth, clarity of presentation, and adequacy of supporting results. The authors are requested to carefully address all reviewer comments and substantially revise the manuscript.

[Note: HTML markup is below. Please do not edit.]

Reviewers' comments:

Reviewer's Responses to Questions

**Comments to the Author**

1. Is the manuscript technically sound, and do the data support the conclusions?

Reviewer #1: Yes

Reviewer #2: Partly

**********

2. Has the statistical analysis been performed appropriately and rigorously?

Reviewer #1: I Don't Know

Reviewer #2: Yes

**********

3. Have the authors made all data underlying the findings in their manuscript fully available?

The PLOS Data policy

Reviewer #1: No

Reviewer #2: Yes

**********

4. Is the manuscript presented in an intelligible fashion and written in standard English?

Reviewer #1: Yes

Reviewer #2: Yes

**********

Reviewer #1: As a reviewer, I have the following comments and questions for the authors to address:

1. The abstract should provide the main outcomes of the study.

2. The novelties and contributions of the study should be clearly stated at the end of the introduction section, highlighting key points.

3. Abbreviations should be defined only once and then used without redefinition.

4. Under STC, the real MPP of the PV panel used is 300W, as shown in Table 1. This differs significantly from the values provided in Table 2; please provide an explanation. The real MPP for each condition should be added in Table 2 for accurate visibility and fair comparison.

5. The predicted power at MPP of the AVSS is greater than that of the proposed AIVSS; however, the MAE of the AIVSS is less than that of the AVSS method. Please clarify this discrepancy.

6. Fig. 7 should be split into two figures for clearer visibility of the obtained results: one should contain the irradiation variation, and the other should contain the power tracking curves.

7. All necessary system parameters used in the experimental validation, along with clear captures or photos, should be provided.

8. As noted in comment 4, under STC, the real MPP of the PV panel used is 300W, as shown in Table 1, which differs significantly from the values shown in Fig. 8. Please provide an explanation.

9. The results presented in Fig. 8 clearly indicate that the AVSS method provides greater power at MPP in comparison with the AIVSS method. However, this is not evident from the comparative data provided in Table 3, which seems to replicate the results in Table 2. A more thorough discussion and commentary on the experimental results are necessary.

10. Relying on a single metric (MAE) is insufficient to prove the accuracy of the proposed method.

11. The discussion of all results should be detailed and rigorous rather than brief and general.

Reviewer #2: An Adaptive Current–Voltage Step Size Solver for Accurate and Efficient Modeling of Solar Photovoltaic Modules

How were the adaptation conditions for λV and λI determined?

The temperature coefficient of voltage (β) is not considered, even though temperature varies. How does this omission affect the MPP accuracy?

What is the purpose of the panel data presented in Section 3.5.2? Please clarify whether it is used in the analysis.

Will the step size ratio of voltage and current remain constant under all irradiance conditions?

What shading patterns and high-temperature values were used under different test conditions? Please specify them clearly.

RMSE analysis is not included in the paper. Please add it to validate the accuracy of the proposed method.

It is not clearly stated whether the proposed method contributes to PV modeling or MPPT tracking. Please clearly distinguish between these two aspects.

**********

what does this mean?). If published, this will include your full peer review and any attached files.

If you choose “no”, your identity will remain anonymous but your review may still be made public.

**Do you want your identity to be public for this peer review?**  For information about this choice, including consent withdrawal, please see our Privacy Policy

Reviewer #1: No

Reviewer #2: No

**********

[NOTE: If reviewer comments were submitted as an attachment file, they will be attached to this email and accessible via the submission site. Please log into your account, locate the manuscript record, and check for the action link "View Attachments". If this link does not appear, there are no attachment files.]

To ensure your figures meet our technical requirements, please review our figure guidelines: https://journals.plos.org/plosone/s/figures

You may also use PLOS’s free figure tool, NAAS, to help you prepare publication quality figures: https://journals.plos.org/plosone/s/figures#loc-tools-for-figure-preparation.

NAAS will assess whether your figures meet our technical requirements by comparing each figure against our figure specifications.

# Author comment: Author response to Decision Letter 1

**DOI:** 10.1371/journal.pone.0359796.r002

21 May 2026

We sincerely thank you for your thorough evaluation and constructive feedback. Your comments have helped us significantly improve the quality, clarity, and rigor of the manuscript. We have carefully revised the paper accordingly. Our detailed responses are provided below.

1. Academic editors

a) PLOS ONE Style Requirements

We carefully reviewed the manuscript and updated it to comply with the PLOS ONE formatting and style requirements, including the use of the appropriate file naming conventions and formatting guidelines provided in the PLOS templates.

b) Code Sharing Requirements

We reviewed the PLOS ONE policy regarding code sharing and reproducibility. The author-generated code underlying the findings of this study has been organized and prepared for public availability in accordance with the journal’s guidelines. Relevant information regarding code access and availability has been included in the manuscript.

c) Use of the PLOS LaTeX Template

The manuscript has been fully updated using the official PLOS LaTeX template as requested. Formatting, bibliography style, figures, tables, and section structures were revised to ensure consistency with PLOS submission requirements.

d) Supporting Information Captions

Captions for all Supporting Information files have now been included at the end of the manuscript. In-text citations referring to the Supporting Information files were also revised to ensure consistency with the captions and journal guidelines.

e) Reviewer-Suggested References

We carefully evaluated all reviewer-recommended references. Relevant publications have been incorporated into the revised manuscript where appropriate. References deemed not directly relevant to the scope or methodology of the present work were not included.

2. Reviewer #1

Comment 1:

The abstract should provide the main outcomes of the study.

Response:

Thank you for this suggestion. The abstract has been revised to explicitly include the key quantitative outcomes of the study. Specifically, we now report the reduction in iteration count (35–40%), improvement in MAE, and computational efficiency gains to better reflect the main findings.

Comment 2:

The novelties and contributions of the study should be clearly stated at the end of the introduction section.

Response:

We agree with this comment. A new paragraph has been added at the end of the Introduction clearly outlining the main contributions of the paper, including:

1. The development of the dual adaptive current–voltage step-size solver (AIVSS),

2. The integration of predictive correction terms,

3. Improved convergence and stability under nonlinear conditions, and

4. Experimental validation under real operating conditions.

Comment 3:

Abbreviations should be defined only once.

Response:

This has been corrected throughout the manuscript. All abbreviations are now defined at their first occurrence and used consistently thereafter without redefinition. (PV, SDM, AVSS, AIVSS)

Comment 4:

Under STC, the real MPP is 300 W, which differs from Table 2.

The reviewer correctly notes the discrepancy between the rated module power (300 W) and the simulated MPP values reported in Table 2. The original simulations were performed using a single-cell equivalent parameter set rather than the full module-scaled parameter set, resulting in lower predicted absolute power values. In the revised manuscript, the SDM parameters have been rescaled to represent the full 60-cell module so that the simulated MPP under STC matches the manufacturer-rated value more closely.

Accordingly, the revised manuscript now includes the reference (rated) MPP values in Table 2 and presents performance evaluation using MAE and RMSE for a more rigorous comparison.

Comment 5:

AVSS shows higher predicted power but higher MAE than AIVSS.

Response:

We appreciate this observation. The higher predicted power of AVSS does not necessarily indicate higher accuracy. In several cases, AVSS slightly overestimates the MPP, leading to larger deviations from the reference values. In contrast, AIVSS provides predictions that are closer to the true/reference MPP, resulting in lower MAE.

This explanation has been clarified in the revised manuscript.

Comment 6:

Fig. 7 should be split.

Response:

This has been implemented. Fig. 7 has been divided into two separate figures:

• One showing irradiance variation

• One showing power tracking response

This improves clarity and readability.

Comment 7:

Provide system parameters and experimental setup photos.

Response:

Additional details of the experimental setup have been included, including:

• Measurement instruments and their specifications

• System parameters

• Sensor descriptions

Furthermore, photographs of the experimental setup have been added to enhance clarity and reproducibility.

Comment 8:

Mismatch between STC (300 W) and Fig. 8 results.

Response:

This issue is related to the explanation provided in Comment 4. The difference arises due to modeling assumptions and operating conditions. The manuscript has been revised to clearly explain this distinction and avoid confusion.

Comment 9:

Inconsistency between Fig. 8 and Table 3.

Response:

We acknowledge this inconsistency. Table 3 has been revised to ensure consistency with the experimental results shown in Fig. 8. Additionally, a more detailed discussion has been included to explain the observed differences between AVSS and AIVSS performance.

Comment 10:

MAE alone is insufficient.

Response:

We agree. Additional performance metrics have been incorporated, including:

• Root Mean Square Error (RMSE)

• Convergence time

• Iteration count

These metrics provide a more comprehensive evaluation of solver performance.

Comment 11:

Discussion should be more rigorous.

Response:

The Results and Discussion section has been substantially expanded. We now include:

• Quantitative comparisons

• Deeper analysis of convergence behavior

• Detailed interpretation of experimental results

• Improved linkage between figures, tables, and conclusions

3. Reviewer #2

Reviewer Comment 1:

How were the adaptation conditions for $\lambda_V$ and $\lambda_I$ determined?

Response:

Thank you for this important observation. We have clarified in the manuscript that the learning coefficients $\lambda_V$ and $\lambda_I$ were selected through sensitivity analysis under varying irradiance and temperature conditions. The revised text now explains that values in the range 0.1 to 0.5 provided the best compromise between convergence speed and numerical stability, while larger values produced oscillatory behavior near the maximum power point.

Revision made: Section 3.3.1 (Adaptive Control Functions)

Reviewer Comment 2:

The temperature coefficient of voltage ($\beta$) is not considered, even though temperature varies. How does this omission affect the MPP accuracy?

Response:

Thank you for this valuable comment. We have clarified that the voltage temperature coefficient ($\beta$) was not explicitly incorporated because the primary objective of this study is to evaluate the numerical performance of the proposed AIVSS solver rather than to develop a detailed temperature-dependent parameter extraction model. Since both AVSS and AIVSS were evaluated under identical modeling assumptions, the comparative assessment of the proposed method remains valid.

Revision made: Section 2.2 (Parameter Dependence)

Reviewer Comment 3:

What is the purpose of the panel data presented in Section 3.5.2? Please clarify whether it is used in the analysis.

Response:

Thank you for pointing this out. We have clarified that the manufacturer module specifications presented in Table 1 serve as reference data for the experimental PV module, while the parameters listed in the “PV Model Parameters” subsection correspond to the extracted single-diode model coefficients used as numerical inputs in the simulations.

Revision made: Section 4.1.2 (PV Model Parameters)

Reviewer Comment 4:

Will the step size ratio of voltage and current remain constant under all irradiance conditions?

Response:

Thank you for this question. We have clarified that the voltage and current step sizes are updated independently according to local power derivatives. Therefore, the ratio $\Delta V/\Delta I$ does not remain constant and varies with irradiance, temperature, and local nonlinearity of the PV characteristic.

Revision made: Section 3.3.3 (Step Size Update Equations)

Reviewer Comment 5:

What shading patterns and high-temperature values were used under different test conditions? Please specify them clearly.

Response:

Thank you for this suggestion. We have now explicitly described the partial shading condition used in the validation, where one-third of the module was subjected to 400 W/m$^2$ while the remaining two-thirds received 1000 W/m$^2$. The temperature conditions were also clarified as 25°C, 50°C, and 75°C for the high-temperature simulations.

Revision made: Section 4.1.1 (Test Conditions)

Reviewer Comment 6:

RMSE analysis is not included in the paper. Please add it to validate the accuracy of the proposed method.

Response:

Thank you for this suggestion. RMSE analysis has now been incorporated throughout the manuscript, including the methodology, performance tables, and discussion section, to provide additional validation of the proposed method’s accuracy.

Revision made: Abstract, Section 5.2, Tables 2 and 4

Reviewer Comment 7:

It is not clearly stated whether the proposed method contributes to PV modeling or MPPT tracking. Please clearly distinguish between these two aspects.

Response:

Thank you for this observation. We have revised the Introduction to clarify that the primary contribution of this work is the improvement of numerical PV module modeling through adaptive dual-step solving, while its application to MPPT is considered a secondary implementation benefit.

Revision made: Introduction section

We sincerely appreciate your insightful comments, which have significantly improved the clarity and technical quality of the manuscript.

Attachment Submitted filename: Response to Reviewers.pdf

# Peer review: Decision Letter 1

**DOI:** 10.1371/journal.pone.0359796.r003

**Authors:** Dinesh Nishad

17 Sep 2026

An Adaptive Current–Voltage Step Size Solver for Accurate and Efficient Modeling of Solar Photovoltaic Modules

PONE-D-25-60979R1

Dear Dr. Mlazi,

We’re pleased to inform you that your manuscript has been judged scientifically suitable for publication and will be formally accepted for publication once it meets all outstanding technical requirements.

Within one week, you’ll receive an e-mail detailing the required amendments. When these have been addressed, you’ll receive a formal acceptance letter and your manuscript will be scheduled for publication.

An invoice will be generated when your article is formally accepted. Please note, if your institution has a publishing partnership with PLOS and your article meets the relevant criteria, all or part of your publication costs will be covered. Please make sure your user information is up-to-date by logging into Editorial Manager at Editorial Manager® and clicking the ‘Update My Information' link at the top of the page. For questions related to billing, please contact billing support.

If your institution or institutions have a press office, please notify them about your upcoming paper to help maximize its impact. If they’ll be preparing press materials, please inform our press team as soon as possible -- no later than 48 hours after receiving the formal acceptance. Your manuscript will remain under strict press embargo until 2 pm Eastern Time on the date of publication. For more information, please contact onepress@plos.org.

Kind regards,

Dinesh Kumar Nishad, PhD

Academic Editor

PLOS One

Additional Editor Comments (optional):

Reviewers' comments:

Reviewer's Responses to Questions

**Comments to the Author**

Reviewer #1: All comments have been addressed

Reviewer #2: All comments have been addressed

**********

2. Is the manuscript technically sound, and do the data support the conclusions?

Reviewer #1: Yes

Reviewer #2: Yes

**********

3. Has the statistical analysis been performed appropriately and rigorously?

Reviewer #1: Yes

Reviewer #2: N/A

**********

4. Have the authors made all data underlying the findings in their manuscript fully available?

The PLOS Data policy

Reviewer #1: No

Reviewer #2: Yes

**********

5. Is the manuscript presented in an intelligible fashion and written in standard English?

Reviewer #1: Yes

Reviewer #2: Yes

**********

Reviewer #1: (No Response)

Reviewer #2: (No Response)

**********

what does this mean?). If published, this will include your full peer review and any attached files.

If you choose “no”, your identity will remain anonymous but your review may still be made public.

**Do you want your identity to be public for this peer review?**  For information about this choice, including consent withdrawal, please see our Privacy Policy

Reviewer #1: No

Reviewer #2: No

**********

# Editor report: Acceptance letter

**DOI:** 10.1371/journal.pone.0359796.r004

**Authors:** Dinesh Nishad

PONE-D-25-60979R1

PLOS One

Dear Dr. Mlazi,

I'm pleased to inform you that your manuscript has been deemed suitable for publication in PLOS One. Congratulations! Your manuscript is now being handed over to our production team.

At this stage, our production department will prepare your paper for publication. This includes ensuring the following:

* All references, tables, and figures are properly cited

* All relevant supporting information is included in the manuscript submission,

* There are no issues that prevent the paper from being properly typeset

You will receive further instructions from the production team, including instructions on how to review your proof when it is ready. Please keep in mind that we are working through a large volume of accepted articles, so please give us a few days to review your paper and let you know the next and final steps.

Lastly, if your institution or institutions have a press office, please let them know about your upcoming paper now to help maximize its impact. If they'll be preparing press materials, please inform our press team within the next 48 hours. Your manuscript will remain under strict press embargo until 2 pm Eastern Time on the date of publication. For more information, please contact onepress@plos.org.

You will receive an invoice from PLOS for your publication fee after your manuscript has reached the completed accept phase. If you receive an email requesting payment before acceptance or for any other service, this may be a phishing scheme. Learn how to identify phishing emails and protect your accounts at https://explore.plos.org/phishing.

If we can help with anything else, please email us at customercare@plos.org.

Thank you for submitting your work to PLOS One and supporting open access.

Kind regards,

PLOS One Editorial Office Staff

on behalf of

Dr. Dinesh Kumar Nishad

Academic Editor

PLOS One
